# Increased circulating progranulin is not sufficient to induce cardiac dysfunction or supraventricular arrhythmia

**DOI:** 10.1038/s41598-023-47311-5

**Published:** 2023-12-06

**Authors:** Kevin E. McElhanon, Tyler C. Huff, Dinesh Hirenallur-Shanthappa, Russell A. Miller, Nicolas Christoforou

**Affiliations:** 1 Rare Disease Research Unit, Worldwide Research, Development, and Medical, Pfizer, Inc., Cambridge, MA USA; 2 Comparative Medicine, Worldwide Research, Development, and Medical, Pfizer, Inc., Cambridge, MA USA

**Keywords:** Cardiomyopathies, Arrhythmias

## Abstract

Atrial fibrillation (AF) is the most prevalent cardiac arrhythmia, and the incidence of new-onset AF has been increasing over the past two decades. Several factors contribute to the risk of developing AF including age, preexisting cardiovascular disease, chronic kidney disease, and obesity. Concurrent with the rise in AF, obesity has followed the same two-decade trend. The contribution of circulating proteins to obesity-related AF is of particular interest in the field. In this study, we investigated the effects of increased circulating levels of the glycoprotein progranulin on the development of supraventricular arrhythmias and changes to cardiac function. AAV8-mediated overexpression of full-length mouse progranulin was used to increase plasma protein levels and determine susceptibility to supraventricular arrhythmias and changes in cardiac structure and function. C57Bl/6N mice were subjected to increased circulating levels of progranulin for 20 weeks. Cardiac conduction was evaluated by surface ECG with and without isoproterenol challenge, and cardiac structure and function were measured by echocardiography after 20 weeks of circulating progranulin overexpression. Increased circulating levels of progranulin were maintained throughout the 20-week study. The cardiac structure and function remained unchanged in mice with increased circulating progranulin. ECG indices (P wave duration, P amplitude, QRS interval) were unaffected by increased progranulin levels and no arrhythmogenic events were observed following the isoproterenol challenge. In our model, increased levels of circulating progranulin were not sufficient to induce changes in cardiac structure and function or elicit ECG abnormalities suggestive of susceptibility to supraventricular arrhythmias.

## Introduction

Atrial fibrillation (AF) is the most common cardiac arrhythmia observed in the clinic. It is estimated that by 2030 approximately 2.1 million individuals will develop AF in the U.S^1,2^. Risk factors for AF include several obesity-related conditions including hypertension, obstructive sleep apnea, underlying cardiomyopathies, left atrial enlargement, and type II diabetes^1–3^. A significant increase in the incidence of AF is observed in individuals 65 years of age and older, and obesity is one of the major driving factors of increased AF in the aging population^4^. AF-associated complications represent a growing health concern with age, leading to increased incidences of myocardial infarction, left atrial thrombus, transition to heart failure, and risk of sudden cardiac arrest^5,6^. The association between obesity and the risk of AF is well established by numerous observational studies^7–10^. While the mechanistic link between obesity and AF remains incompletely understood, several obesity-related pathophysiological factors including left atrial enlargement, epicardial adipose tissue volume increases, and adipokine signaling contribute to AF pathogenesis and progression (reviewed in^11–13^). Numerous circulating proteins involved in inflammation and fibrosis are associated with paroxysmal and/or persistent AF^14^. Notably, several of these proteins include various adipokines such as adiponectin and omentin and interleukins secreted from adipose tissue^15–17^.

Progranulin is a highly conserved secreted glycoprotein expressed in numerous cell types and has recently been identified as a novel adipokine associated with obesity and insulin resistance^18^. The function of progranulin in cardiac tissue and its role in cardiovascular disease remains to be fully elucidated. A protective role of recombinant progranulin in acute myocardial ischemia/reperfusion injury has been reported^19,20^, while cleavage of full-length progranulin into granulin peptides is thought to contribute to inflammation in atherosclerotic plaques contributing to atherosclerosis^21,22^. Elevated levels of progranulin have also been associated with incident heart failure, all-cause mortality, and cardiovascular disease mortality^23,24^. Recently, serum levels of progranulin have been observed to increase preceding adverse cardiac events in individuals with heart failure, with the most significant increases observed in individuals with diabetes and atrial fibrillation as comorbidities^25^. Although full-length progranulin is mainly thought to be anti-inflammatory, the pro- versus the anti-inflammatory effect of progranulin may vary in a tissue-specific or pathology-specific manner. Several studies have identified increased circulating levels of progranulin in obesity and type II diabetes^26–28^. Interestingly, progranulin knock-out mice are protected from insulin resistance and obesity when fed a high-fat diet and have reduced levels of IL-6^18^. This finding suggests the possibility of a causal chain linking obesity, increased circulating levels of progranulin, and the risk of AF susceptibility. In the present study, we tested the hypothesis that increases in circulating progranulin represent a potential causal event in the development of AF and/or result in adverse cardiac remodeling and decreased cardiac function.

## Results

### AAV8-Grn dose optimization

Genome-wide meta-analyses and clinical studies have identified obesity and type 2 diabetes as drivers of increased circulating levels of progranulin^26–28^. To further investigate the association between increased circulating progranulin and the risk of AF, we used AAV serotype 8 to increase the expression of full-length progranulin in the liver. To determine the optimal doses of the AAV8-Grn vector, C57Bl/6N mice received increasing doses (1.2 × 10^11^, 4.0 × 10^11^, 1.2 × 10^12^, 4.0 × 10^12^, and 1.2 × 10^13^ VG/kg) of an AAV8 vector driving expression of mouse progranulin under the control of a liver-specific TBG promoter. Doses of 1.25 × 10^12^ VG/kg (hereafter referred to as Grn Low) and 4.17 × 10^12^ VG/kg (hereafter referred to as Grn High) were selected for subsequent studies. 1.25 × 10^12^ VG/kg recapitulated the approximately twofold increase reported in obese individuals^27^. 4.17 × 10^12^ VG/kg represents a supraphysiological increase in progranulin levels ≥ a fivefold increase in circulating progranulin (Supplemental Fig. S1). Our experimental design utilized overexpression of progranulin with the liver-specific TGB promoter, therefore liver toxicity was evaluated by measuring plasma levels of alanine transaminase (ALT) and aspartate aminotransferase (AST). No changes in liver toxicity markers were observed at any of the AAV8- Grn doses tested. Several studies have identified increased circulating levels of progranulin in obesity and type II diabetes^26–28^, however, no changes were observed in the circulating analytes β-hydroxybutyric acid (BHBA), cholesterol, non-fasted glucose, or circulating triglyceride levels (Supplemental Fig. S2).

### AAV8-Grn bGH maintains prolonged elevation of circulating progranulin levels

**Figure 1:**
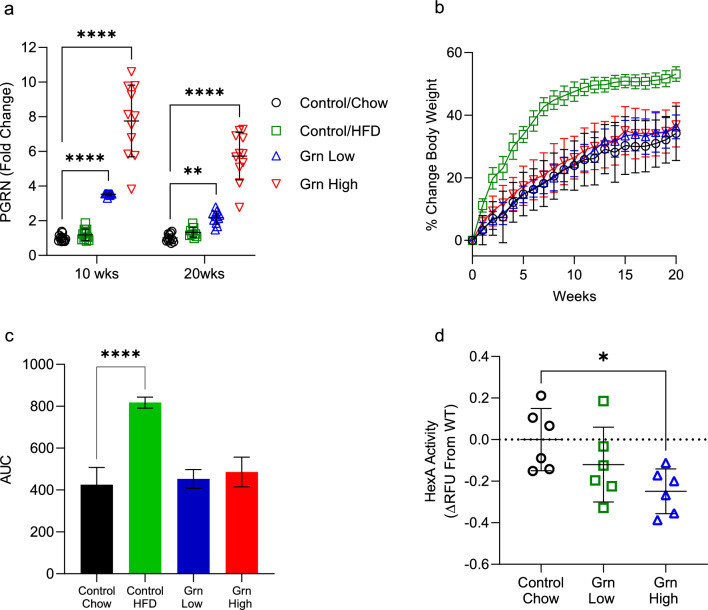
AAV8-Grn bGH vector results in significantly elevated levels of circulating progranulin 20 weeks post-injection. Mice received retro-orbital injections of AAV8-Grn bGH vector at 1.25 × 10^12^ (Grn Low) and 4.17 × 10^12^ (Grn High) VG/kg and plasma levels of progranulin quantified by ELISA. (**a**) Plasma progranulin levels 10 weeks post-injection (Control/Chow and Grn Low, n = 12, Control/HFD, and Grn High, n = 11). Plasma progranulin levels 20 weeks post-injection (Control/Chow and Grn High, n = 11; Control/HFD, n = 9; Grn Low, n = 12). (**b**) % Change in body weight measured weekly for 20 weeks. (**c**) Area under the curve of (**b**). (**d**) Hex A activity measured in liver homogenates from controls and progranulin overexpressing mice with the fluorogenic substrate 4-methylumbelliferyl-6-sulfo-N-acetyl-beta-D-glucosaminide (n = 6). *p = 0.02, **p = 0.0023, ****p < 0.0001. Data are presented as individual values, bars represent mean ± SD.

10 weeks post-injection of AAV8-Grn bGH, plasma levels of progranulin were quantified by ELISA and found to be increased 3.5-fold (Grn Low) and 7.75-fold (Grn High) versus empty vector controls (Fig. 1a). At the 20-week endpoint, plasma levels were slightly decreased from 10 weeks (Grn Low, 2.16-fold; Grn High, 5.2-fold increase), however, our target expression levels were maintained throughout the 20-week study (Fig. 1a). The percent change in body weight was monitored weekly for 20 weeks and no significant changes were observed in progranulin overexpressing mice versus empty vector controls on normal chow (Fig. 1b,c). To determine if the AAV8-Grn vector produces a functional progranulin protein, β-hexosaminidase A (Hex A) activity was measured using the fluorogenic substrate 4-Methylumbelliferyl-6-sulfo-N-acetyl-beta-D-glucosaminide. Progranulin insufficiency has been demonstrated to cause an increase in Hex A activity in patients with frontotemporal dementia and increased Hexa mRNA levels in hepatocytes isolated from mice fed a high-fat diet^29,30^. To our knowledge, a specific assay to measure progranulin activity is not available, therefore, we quantified Hex A activity in mice overexpressing progranulin as an indirect measure of progranulin activity. Liver-directed overexpression of progranulin led to a decrease in Hex A activity in Grn High mice (Fig. 1d; ΔRFU relative to control: − 0.12 ± 0.18, and − 0.25 ± 0.11 for Grn low and Grn high respectively).

### Increased circulating progranulin does not affect cardiac structure or function

**Figure 2:**
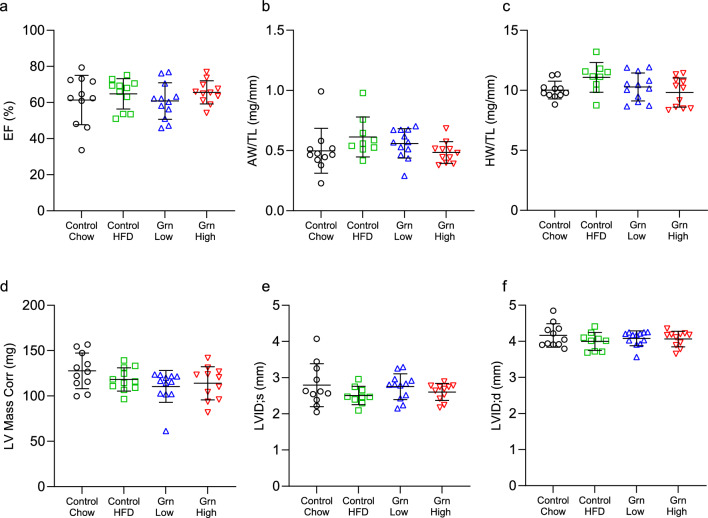
Chronic overexpression of circulating progranulin does not alter cardiac structure or function. After 20 weeks of progranulin overexpression, echocardiography was performed on all groups to determine systolic function and degree of left ventricular remodeling. Progranulin overexpression does not alter: (**a**) left ventricular ejection fraction, (**b**) atria weight/tibia length, (**c**) heart weight/tibia length, (**d**) left ventricular mass, (**e**,**f**) or left ventricular internal diameter at systole and diastole. Control/Chow n = 11, Control/HFD n = 9, Grn Low n = 12, and Grn High n = 11. p > 0.05 for all comparisons. Data are presented as individual values, bars represent mean ± SD.

Plasma levels of progranulin have been observed to increase before adverse cardiac events and are negatively associated with clinical outcomes in heart failure patients, however, if this elevation is a cause or a consequence of disease progression remains to be elucidated^25^. To determine if elevated plasma progranulin levels negatively impact cardiac structure and function changes to the left ventricular structure and ejection fraction were evaluated by echocardiography after 20 weeks of progranulin overexpression. Ejection fraction was unaffected by 20 weeks of elevated progranulin or due to diet-induced obesity (Fig. 2a). Adverse cardiac remodeling in the form of LV internal diameter (Fig. 2e,f) and anterior/posterior wall thickness (Supplementary Table S2) was also unaffected by progranulin overexpression or diet-induced obesity. Additionally, atria weight/tibia length, heart weight/tibia length ratios, and left ventricular mass (Fig. 2b–d) remained unchanged in all groups. Changes in progranulin protein levels were also evaluated in cardiac tissue by immunoblot and no significant elevations were observed for any treatment group, consistent with progranulin being a constitutively secreted protein (Supplemental Fig. S3a,b).

### Increased circulating progranulin does not alter interatrial or atrioventricular conduction

**Figure 3:**
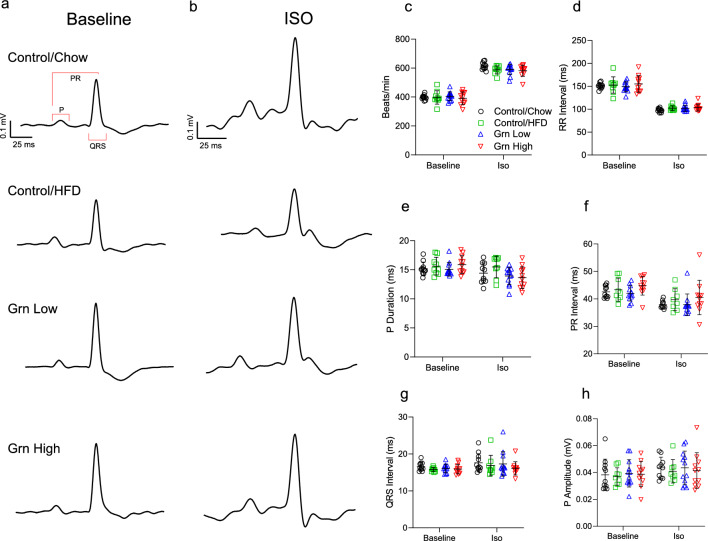
Chronic overexpression of circulating progranulin does not affect cardiac electrical activity. At 20 weeks chronic progranulin overexpression surface ECGs were recorded to quantify cardiac electrical activity. Baseline measures represent averaged values from a 5-min recording under 1.5% isoflurane. Following baseline, mice received a 3mg/kg bolus of the β-adrenergic agonist isoproterenol. (**a**,**b**) Representative traces of one cardiac cycle at baseline (**a**) and following isoproterenol challenge (**b**). (**c**) Chronotropic response to isoproterenol challenge (beats/minute). (**d**) RR interval. (**e**) Atrial conduction time (P duration). (**f**) PR interval. (**g**) QRS interval. (**h**) P wave amplitude. Control/Chow n = 11, Control/HFD n = 9, Grn Low n = 12, and Grn High n = 11. p > 0.05 for all comparisons. Data are presented as individual values, bars represent mean ± SD.

Clinical studies have linked elevated progranulin with AF risk in patients with underlying cardiomyopathies and the pro-inflammatory properties of progranulin have been linked with a risk of cardiovascular disease^24,25,31^. Additionally, plasma progranulin levels are known to increase in obesity^18,27^. Prolonged P wave duration and a shortening of the PR interval have been observed in diet-induced obesity mouse models of AF^32,33^. To determine if elevated plasma progranulin represents a causal event leading to atrial conduction abnormalities, surface ECG was performed at the 20-week timepoint. Following baseline surface ECG recordings, cardiac stress was induced with an IP bolus (3 mg/kg) of isoproterenol (ISO), and ECG traces were recorded for an additional 10 min. Representative traces depicting one cardiac cycle at baseline and following ISO challenge are depicted in Fig. 3a,b. ISO challenge elicited a significant increase in heart rate and a decrease in RR interval, indicating an appropriate chronotropic response in all groups (Fig. 3c,d). At baseline, P wave duration, P amplitude, and PR interval were unaffected by elevated progranulin levels or obesity (Fig. 3e,f,h). As per baseline ECG indices, no changes were observed in P wave duration, PR interval, or QRS interval following the ISO challenge (Fig. 3e–h).

**Figure 4:**
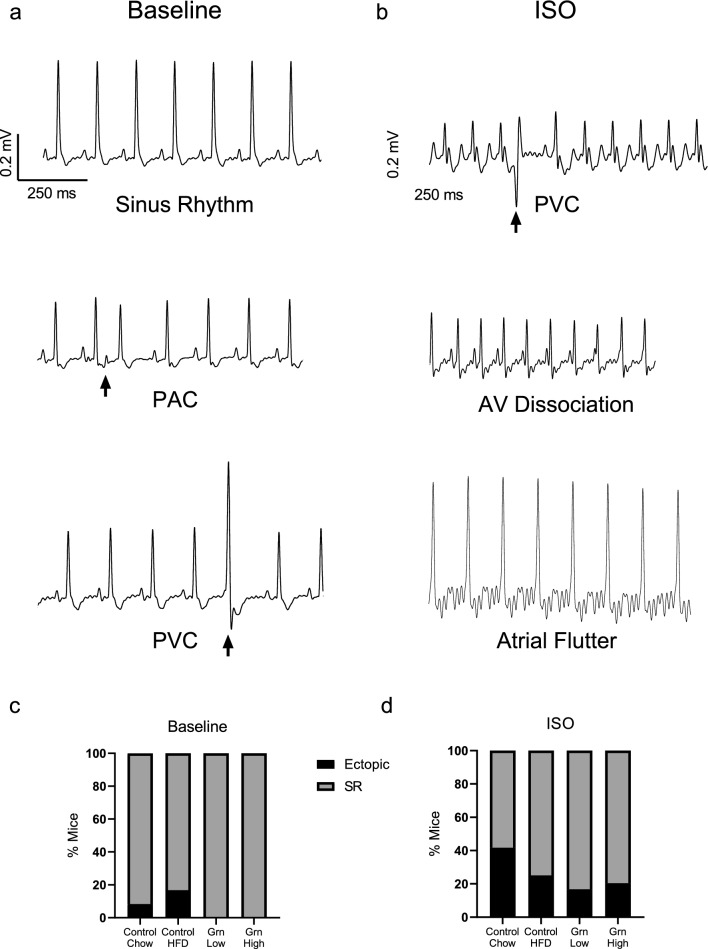
20 weeks of elevated plasma progranulin does not result in increased ectopic beats/cardiac rhythms. (**a**,**b**) Representative ECG traces of ectopic events at baseline (**a**) and following isoproterenol challenge (**b**) irrespective of treatment group. (**c**) Percentage of mice with ectopic beats/cardiac rhythms at baseline. (**d**) Percentage of mice with ectopic beats/cardiac rhythms following isoproterenol challenge. *SR* sinus rhythm, *PVC* premature ventricular contraction, *PAC* premature atrial contraction, *AV Dissociation*: atrioventricular dissociation.

ECG traces at baseline and following the ISO challenge were manually analyzed for instances of ectopic beats and instances of arrhythmia in all groups. Ectopic beats were defined as premature ventricular contractions (PVC, lack of discernable p wave preceding QRS with shortened RR interval), or premature atrial contractions (PAC, presence of P wave with shortened P-P interval and RR interval). Episodes of abnormal cardiac rhythm were defined as irregularly irregular RR intervals or aberrant atrial conduction in the form of independent atrial and ventricular contractions (atrioventricular dissociation) or a sawtooth pattern of atrial excitation preceding each QRS complex (atrial flutter) lasting ≥ 1 s in duration. Representative traces of ectopic beats and aberrant atrial conduction are depicted in Fig. 4a,b irrespective of treatment group. No instances of arrhythmia or ectopic beats were observed in Grn Low or Grn High mice at baseline versus 8.3% and 16.7% of empty vector control mice on chow or HFD respectively (Fig. 4c and Table 1). Following ISO challenge ectopic events increased in all groups with most events observed in control groups (41.7% control/chow, 25% control/HFD versus 16.7% Grn Low, 20% Grn High (Fig. 4d and Table 1).

**Table 1:** Incidence of ectopic beats/rhythms.

	Ectopic events				
	**n**	PVC	PAC	Atrial flutter	AV diss
Baseline					
Control/chow	11	4 (1)	–	–	–
Control/HFD	9	–	2 (2)	–	–
Grn low	12	–	–	–	–
Grn high	11	–	–	–	–
ISO					
Control/chow	11	18 (3)	2 (2)	–	1 (1)
Control/HFD	9	2 (2)	–	–	1 (1)
Grn low	12	1 (1)	–	1 (1)	1 (1)
Grn high	11	7 (2)	–	–	–

Data represents # ectopic events (# mice/group).

*PVC* premature ventricular contraction, *PAC* premature atrial contraction, *AV diss.* atrioventricular dissociation.

### Increased circulating progranulin is not associated with the expression of inflammatory or fibrosis-related genes

**Figure 5:**
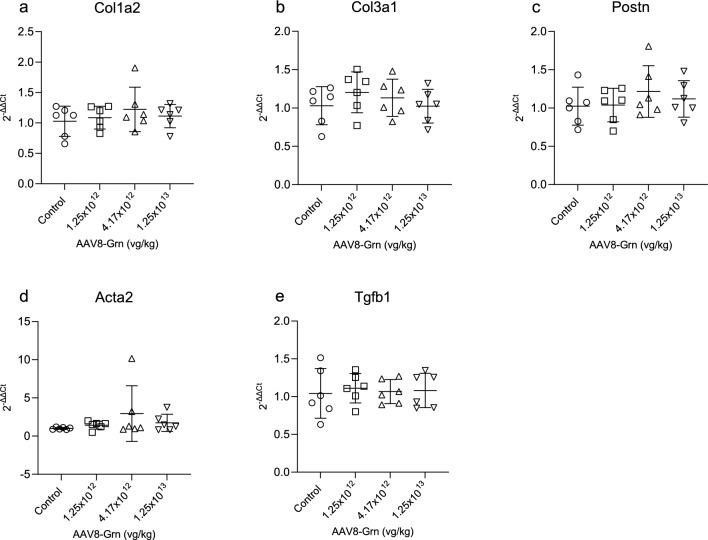
Elevated levels of circulating progranulin do not alter the expression of fibrosis-related genes. RNA was isolated from the atria of control mice and mice receiving AAV8-Grn bGH vector at indicated doses 6 weeks post-injection and markers of fibrosis was evaluated by qPCR. No changes were observed in mRNA levels of (**a**) Col1a2, (**b**) Col3a1, (**c**) Postn, (**d**) Acta2, and (**e**) Tgfb1. n = 6 for all groups. p > 0.05 for all comparisons. Data are presented as individual values, bars represent mean ± SD.

**Figure 6:**
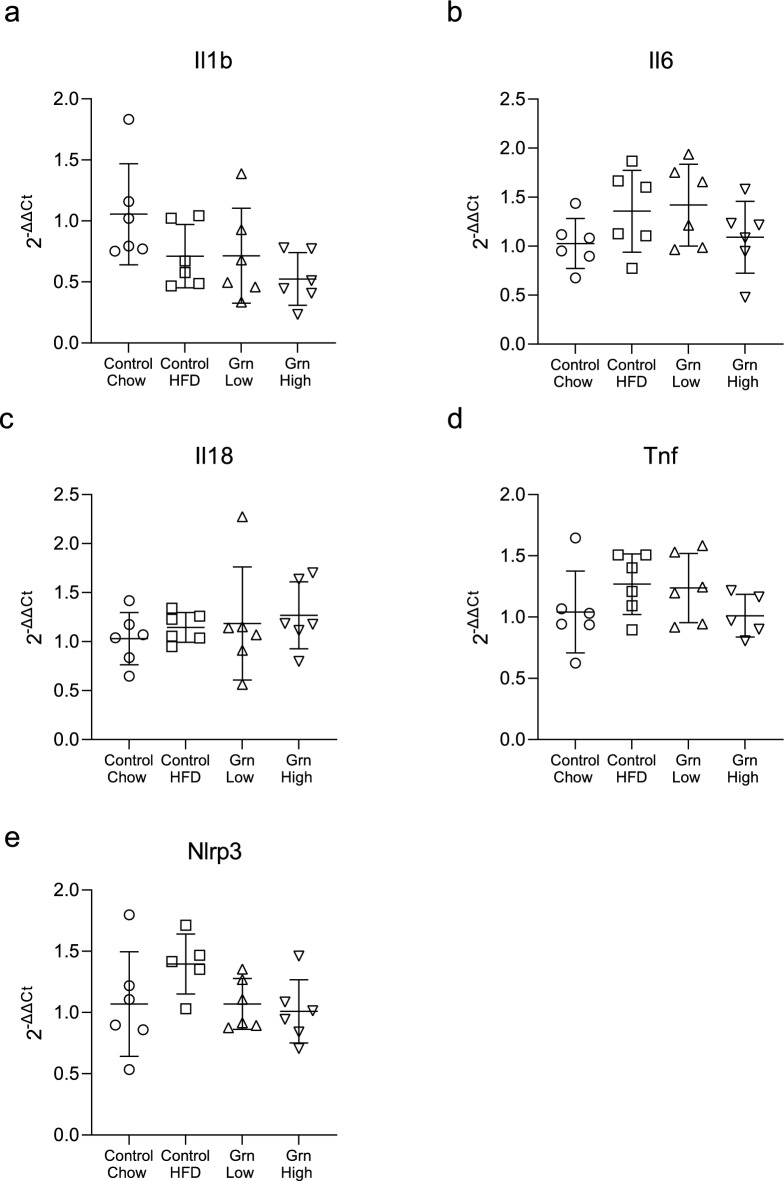
Increased circulating progranulin does not affect atrial expression levels of pro-inflammatory genes. RNA was isolated from the atria of all groups at 20 weeks and expression levels of pro-inflammatory genes evaluated by qPCR. No significant changes in expression levels of Il1b, Il6, Il18, Tnf, or Nlrp3 were observed (**a**–**e**). n = 6 for all groups. p > 0.05 for all comparisons. Data are presented as individual values, bars represent mean ± SD.

Atrial fibrosis is one of the major pathologies driving the onset of AF and growing evidence implicates the involvement of obesity and adipokine signaling in atrial remodeling creating pro-AF substrate^11,13,33,34^. Progranulin levels are known to increase in obesity and have been demonstrated to mediate TNF induced increases in the proinflammatory cytokine IL-6 and insulin resistance^18^. Insulin resistance has been linked to an increased risk of AF and TGF-β1 and collagen expression is increased in the atria of obese rats, suggesting progranulin may have a role in atrial fibrosis^34^. mRNA levels of pro-fibrotic markers were evaluated by qPCR in the atria from mice in the dose optimization study, allowing for the determination of gene expression at our experimental doses and an additional, higher dose of 1.25 × 10^13^ VG/kg. No changes were observed in mRNA levels of Col1a2, Col3a1, Acta2, Tgfβ1, or Postn in progranulin overexpressing mice versus controls (Fig. 5a–e). Importantly, AAV8-Grn bGH at a dose of 1.25 × 10^13^ VG/kg, resulting in a greater than 40-fold increase in plasma progranulin, did not increase fibrosis-related gene expression (Fig. 5a–e and Supplemental Fig. S1b). Systemic and local inflammation have been demonstrated to play a critical role in the development of an arrhythmic substrate^35,36^. We next evaluated the effect of increased circulating progranulin on inflammatory gene expression in atrial tissue after 20 weeks of overexpression. No significant differences were observed in the mRNA levels of Il1b, Il6, Il8, Tnf, or Nlrp3 (Fig. 6a–e).

## Discussion

A previously reported observational study identified temporal increases in serum progranulin preceding adverse cardiac events in heart failure patients, with AF and type II diabetes being among the strongest determinants of progranulin levels in the parameters tested^25^. To test the potential causal association of increased circulating progranulin and the risk of developing AF, an AAV serotype 8 viral vector containing cDNA for the full-length mouse progranulin gene driven by the liver-specific TBG promotor was used to increase circulating levels of progranulin. Elevated levels were verified by ELISA at 10 weeks and 20 weeks and confirmed our experimental model sufficiently elevated circulating progranulin above physiological levels and were maintained for the 20-week duration of the study. However, elevated plasma levels of progranulin did not adversely affect cardiac function or structure or lead to supraventricular arrhythmia at the levels achieved in this study.

A potential explanation for the increased circulating levels of progranulin observed in obesity and the association with adverse cardiac events could be due to bidirectional causality or confounding of reduced renal function^37,38^. The association between obesity and reduced kidney function is well established and AF and chronic kidney disease share common obesity-related risk factors including diabetes, hypertension, and atherosclerosis^39^. Obesity is an independent risk factor for decreased estimated glomerular filtration rate (eGFR) and serum levels of progranulin increase with decreased renal function^40^. Interestingly, a genome-wide meta-analysis of circulating cardiovascular proteins found that BMI and eGFR causally affected plasma levels of 37.6% and 34.1% respectively of the 90 proteins tested^41^. The major route of progranulin elimination is renal filtration^40,42^, therefore, decrease renal function associated with the obese state, AF, type II diabetes, and heart failure may explain the observed increases in progranulin levels.

Interestingly, some studies have demonstrated the protective effect of progranulin in cardiac tissue. Administration of recombinant progranulin prior to or immediately following myocardial ischemia/reperfusion injury in rats resulted in an increased recovery of cardiac contractility, significantly reduced creatine kinase and LDH levels, reduced inflammation, and increased expression of the anti-apoptotic protein Bcl-2^19^. Similar protective effects have been observed in permanent left coronary artery occlusion in mice and rabbits treated with recombinant progranulin^20^. In both models, administration of recombinant progranulin before LCA occlusion in mice and myocardial ischemia/reperfusion injury in rabbits reduced leukocyte infiltration and infarct size. The protective effects of progranulin have also been observed in hyperhomocysteinemia-induced cardiorenal injury via negative regulation of Wnt/B-catenin signaling in the renal cortex^43^.

In each of these studies, recombinant progranulin was administered prophylactically before or during the experimental insult or using progranulin-deficient mice. Initiation and termination of the inflammatory response are tightly and temporally regulated to maintain physiological homeostasis^44^. Modulation of the immune response and inflammation by progranulin and its granulin cleavage products is well documented (reviewed in^45^). While limited data is available regarding local progranulin function in cardiac tissue, AAV-GRN overexpression in the brains of Grn null mice has been shown to lead to a local inflammatory response^46^ and neuronal toxicity^47^ that was not seen in AAV-GFP control animals. In this study, we set out to determine if chronic increases in circulating progranulin represent a causal event leading to adverse cardiac remodeling and/or aberrant atrial conduction, potentially representing an intermediate condition leading to AF pathogenesis. It is important to note that in this study, we tested the effect of increased circulating progranulin in the absence of any cardiac insult. Our goal was to determine if increased circulating progranulin represents a causal event leading to arrhythmias or cardiac dysfunction. Additionally, we did not observe increased progranulin levels in cardiac tissue using our liver-directed overexpression approach. In conclusion, our data indicate that 20 weeks of elevated circulating progranulin is well tolerated in mice and does not impact cardiac structure or function.

## Clinical significance and study limitations

Progranulin is involved in cell growth, promotes wound healing, regulates lysosome function, and has a protective role in neuronal degradation^48–53^. Various proteases can cleave full-length progranulin to form eight granulin peptides with pro-inflammatory actions, while the intact precursor is thought to confer an anti-inflammatory effect^54^. Diseases associated with mutations in the progranulin gene include frontotemporal dementia due to haploinsufficiency and homozygous mutations are known to result in neuronal ceroid lipofuscinosis^55^. Progranulin gene replacement has become an attractive therapeutic target in recent years for the treatment of frontotemporal dementia^56^. Here, we demonstrate that 20 weeks of elevated circulating progranulin does not adversely affect cardiac structure or function.

It is important to note that in this study we evaluated the effect of increased circulating progranulin on cardiac function in the absence of cardiac insult. While supraphysiological increases in progranulin were well tolerated in healthy animals, no conclusions can be made concerning a protective, or exacerbating effect on preexisting cardiomyopathies. Additionally, our analysis of arrhythmia susceptibility did not include a programmed electrical atrial burst pacing protocol, which is typically used to induce atrial fibrillation in mice.

## Materials and methods

### Mice

All work with animals was performed in compliance with the ARRIVE guidelines and in accordance with the recommendations of the NIH Guide for the Care and Use of Laboratory Animals^57,58^. Experimental procedures were reviewed and approved by the Pfizer Institutional Animal Care and Use Committee and conducted in an Association for Assessment and Accreditation of Laboratory Animal Care (AAALAC) accredited facility. 7–8-week-old male C57Bl/6N mice were purchased from Taconic Biosciences (Germantown, NY) for all in vivo studies. Mice were randomly assigned to the treatment groups as detailed below. All mice were housed at standard conditions with a 12-h on/12-h off light cycle and provided ad libitum access to drinking water and chow.

### AAV8 constructs

Long-term elevation of circulating progranulin was achieved using AAV serotype 8 (AAV8) to deliver cDNA encoding the full-length Mus musculus progranulin gene (Gene ID: 14824) with a liver-specific thyroxine-binding globulin (TBG) promoter and bovine growth hormone (bGH) poly(A) sequence. AAV8-Grn bGH and empty control vectors were purchased from the University of Massachusetts Medical School Gene Therapy Center Vector Core (Worcester, MA).

### AAV8-Grn dose optimization

To determine optimal doses of AAV8-Grn bGH vector resulting in a physiological twofold increase in circulating progranulin observed in obese individuals with BMI ≥ 25^35^ and a supraphysiological increase, defined as a ≥ fivefold increase, male C57Bl/6N mice were randomly assigned to treatments groups (n = 6/group) and received retro-orbital injections of sterile saline or AAV8-Grn bGH vector at doses of 1.2 × 10^11^, 4.0 × 10^11^, 1.2 × 10^12^, 4.0 × 10^12^, and 1.2 × 10^13^ VG/kg in a total volume of 100 µL. 6-weeks post-injection mice were euthanized, and whole blood was collected by cardiac puncture. Plasma levels of progranulin were quantified by ELISA and doses of 1.25 × 10^12^ and 4.17 × 10^12^ VG/kg were selected for subsequent studies.

### Increased plasma progranulin expression

Male C57Bl/6N mice were randomly divided into four treatment groups (n = 12/group). Control/Chow mice received retro-orbital injections of empty AAV8 vector and were maintained on a normal chow diet for the duration of the study. Control/High-fat diet (HFD) mice received empty vectors and were placed on a high-fat diet with 60 kcal% fat (Research Diets, D12492). Progranulin overexpressing mice received AAV8-Grn bGH vector at doses of 1.25 × 10^12^ (Grn Low) or 4.17 × 10^12^ VG/kg (Grn High) and received normal rodent chow for the duration of the study. As detailed below, plasma levels of progranulin were evaluated at 10 weeks and 20 weeks post-injection.

### Circulating progranulin quantification

Whole blood was collected by tail snip or terminal cardiac puncture. Plasma was isolated from whole blood by centrifugation and levels of progranulin were quantified using the R&D Systems Quantikine ELISA, Mouse Progranulin ELISA (Cat# MPGRNO) following the manufacturer’s protocol at dilutions of 1:200 (AAV8 empty vector) or 1:1500 (4.0 × 10^12^ and 1.2 × 10^13^ VG/kg AAV8-Grn bGH) to stay within the linear range of the assay. Standard curves and interpolated plasma concentrations of progranulin were calculated using GraphPad Prism 9.0.0 with a sigmoidal, 4PL least squares fit.

### RNA Isolation and qPCR

Flash-frozen atria were harvested following dose optimization and after 20 weeks of progranulin overexpression. A homogenized in 1 mL Qiazol with 200 µL chloroform in 2 mL Matrix D Lysis Tube with the FastPrep 24 TG bench top bead beating system (MP Biomedicals) using the preset lysis protocol for murine heart tissue. RNA was isolated from homogenates with Qiagen RNEasy spin columns and reagents following the manufacturer’s protocol. Quantification of fibrosis gene markers by qPCR used the TaqMan probes in Supplemental Table S1 with the TaqMan RNA-to-Ct 1-Step Kit (Life Technologies, 4392653).

### Hexosaminidase A activity assay

To determine if the over-expression of progranulin with the AAV8-Grn bGH vector induced transcription of a functional protein, hexosaminidase A (Hex A) activity was determined with the fluorogenic substrate 4-Methylumbelliferyl-6-sulfo-N-acetyl-beta-D-glucosaminide^37,38^ (Millipore Sigma, 454428). Briefly, 25 µg of liver tissue lysate was added to 25 µL assay buffer (0.2 M NaOAc, pH 4.0 with 3 mM 4-Methylumbelliferyl-6-sulfo-N-acetyl-beta-D-glucosaminide substrate) and incubated for 30 min at 37 °C. 75 µL of 1 M glycine, pH 10 was then added to stop the reaction and fluorescence measured with an Envision plate reader (PerkinElmer) with an excitation wavelength of 355 nm and emission wavelength of 450 nm.

### Protein extraction and immunoblotting

Approximately 10 mg of flash-frozen heart tissue were placed in 2 mL lysis matrix D tubes with 500 µL RIPA buffer plus protease and phosphatase inhibitors. Tissue samples were homogenized with the FastPrep 24 TG bench top bead beating system (MP Biomedicals) using the preset lysis protocol for murine heart tissue. Lysates were cleared of debris by centrifugation at 18,000×*g* for 20 min and protein concentration was determined by BCA protein assay. Antibodies used for immunoblotting include Granulins Polyclonal Antibody (Invitrogen, PA5-27275), Goat anti-Rabbit IgG (Abcam, ab6721), and anti-GAPDH (D16H11)XP Rabbit mAb (HRP conjugate) (Cell Signaling, 8884). 35 µg of total protein were loaded and separated on NuPAGE 4–12% Bis–Tris, 1.0 mm Mini Protein Gels (Invitrogen) at 200 V constant. Proteins were then transferred to nitrocellulose membranes at 30 V constant for 60 min. Membranes were blocked for 60 min with 5% casein in Tris-buffered saline with Tween-20 (TBST) with gentle agitation, incubated with primary antibodies (1:5000) overnight, followed by washing 3X and 60-min incubation with secondary antibodies. Chemiluminescence image capture was performed with the Amersham ImageQuant 800 using the automatic exposure setting to prevent saturation of bands of interest.

### Echocardiography

Assessment of cardiac structure and function was evaluated by echocardiography with the Vevo 3100 Preclinical Imaging System (VisualSonics) with an MX550D scan head after 20 weeks of progranulin overexpression. Parasternal short-axis cine loops were acquired from anesthetized mice under 1–1.5% sevoflurane for analysis of ejection fraction, fractional shortening, left ventricular internal diameter, and anterior/posterior left ventricular wall thickness at diastole and systole as previously described^59^.

### Surface ECG

ECG indices and arrhythmogenic events were assessed following 20 weeks of elevated circulating progranulin levels. Mice were anesthetized under 2% isoflurane and proper induction was verified by toe pinch. Mice were then placed in a supine position on a heated stage maintained at 37 °C and isoflurane was continuously delivered via nose cone. Needle electrodes were inserted subcutaneously in a lead 1 configuration (left foreleg, right foreleg, and left hindleg). Isoflurane was then adjusted to 1.5% and the heart rate was allowed to stabilize before baseline recordings. A 5-min baseline ECG recording was acquired, followed by a 3 mg/kg intraperitoneally injected bolus of the β-adrenergic receptor agonist isoproterenol (Sigma-Aldrich, I6504) and an additional 10 min of ECG traces recorded. Analysis of P wave duration, P wave amplitude, PR interval, and QRS interval was quantified using the ECG Analysis Module in LabChart 8. For baseline ECG analysis, the entire 5-min baseline recording was averaged to generate a representative trace, and values were determined using the automated ECG analysis tool with manual adjustment of measurements as appropriate. ECG analysis after the isoproterenol challenge was performed as per baseline, with 10 min of the ECG recording used for analysis after heart rate stabilization following the isoproterenol challenge. Identification of ectopic beats and/or arrhythmogenic events was performed manually using the entire recording (5-min baseline and 10 min post-isoproterenol injection).

### Statistical analysis

Graphical representation and statistical analysis of data was performed using GraphPad Prism version 9.0. Data were analyzed by one-way ANOVA or Mixed-effects model. Post hoc analysis was performed with Tukey’s HSD or Dunnett’s multiple comparisons tests as appropriate. An adjusted p-value ≤ 0.05 was determined as statically significant.

### Supplementary Information

Supplementary Information.

## Data Availability

The datasets generated and analyzed in this study are available from the corresponding author upon reasonable request.
