# Supplementary material for: Increased circulating progranulin is not sufficient to induce cardiac dysfunction or supraventricular arrhythmia
Source: Sci Rep. 2023 Dec 6;13:21541. doi: 10.1038/s41598-023-47311-5 (PMC10700350; doi:10.1038/s41598-023-47311-5)

Supplemental Material:

Supplemental Table S1: TaqMan Probes for qPCR

Gene Symbol	Gene Name	Assay ID
Postn	periostin	Mm00450111_m1
Acta2	actin, alpha 2, smooth muscle	Mm00725412_s1
Col1a2	collagen, type I, alpha 2	Mm00483888_m1
Col3a1	collagen, type III, alpha 1	Mm01254476_m1
Tgfb1	transforming growth factor, beta 1	Mm01178820_m1
Il1b	Interleukin 1 beta	Mm00434228_m1
Il6	Interleukin 6	Mm00446190_m1
Il18	Interleukin 18	Mm00434226_m1
Tnf	Tumor necrosis factor	Mm00443258_m1
Nlrp3	NLR family, pyrin domain containing 3	Mm00840904_m1

Supplemental Table S2: Echocardiography Measures

		HR	LVID; s	LVID; d	SV	EF	FS	CO	LV Mass Cor	LV AW; s	LV AW; d	LV PW; s	LV PW; d
**Diet**	**Vector**	**BPM (±SD)**	**Mm (±SD)**	**Mm (±SD)**	**µL (±SD)**	**% (±SD)**	**% (±SD)**	**mL/min (±SD)**	**Mg (±SD)**	**Mm (±SD)**	**Mm (±SD)**	**Mm (±SD)**	**Mm (±SD)**
NC	Empty	542.8 (21.4)	2.8 (0.6)	4.2 (0.3)	46.2 (7.3)	61.4 (13.7)	33.4 (9.4)	25.0 (3.7)	127.8 (19.6)	1.5 (0.1)	1.0 (0.1)	1.2 (0.2)	0.9 (0.1)
HFD	Empty	529.6 (19.2)	2.5 (0.3)	4.0 (0.2)	47.5 (8.1)	67.6 (6.3)	37.3 (4.7)	25.1 (4.1)	117.6 (12.9)	1.6 (0.1)	1.0 (0.1)	1.3 (0.2)	0.9 (0.1)
NC	Grn Low	534.7 (18.1)	2.8 (0.4)	4.1 (0.2)	44.7 (7.9)	60.9 (10.1)	32.7 (7.4)	23.9 (4.2)	110.5 (17.5)	1.4 (0.2)	0.8 (0.1)	1.3 (0.2)	0.9 (0.1)
NC	Grn High	536.2 (20.5)	2.6 (0.2)	4.1 (0.2)	48.0 (7.5)	65.6 (6.4)	35.9 (5.0)	25.7 (4.2)	114.0 (18.3)	1.6 (0.1)	1.0 (0.1)	1.2 (0.1)	0.8 (0.1)

Values in the table are presented as units (± standard deviation). Chow/Empty n=11; HFD/Empty n=9; Chow/Grn Low n=12; Chow/Grn High n=11. Abbreviations: NC (normal chow); HFD (high-fat diet); HR (heart rate); LVID;s/d (left ventricular internal diameter; systole/diastole); SV (stroke volume); EF (ejection fraction); FS (fractional shortening); CO (cardiac output); LV Mass Cor (left ventricular mass corrected); LVAW;s/d (left ventricular anterior wall thickness; systole/diastole); LVPW;s/d (left ventricular posterior wall thickness; systole/diastole).

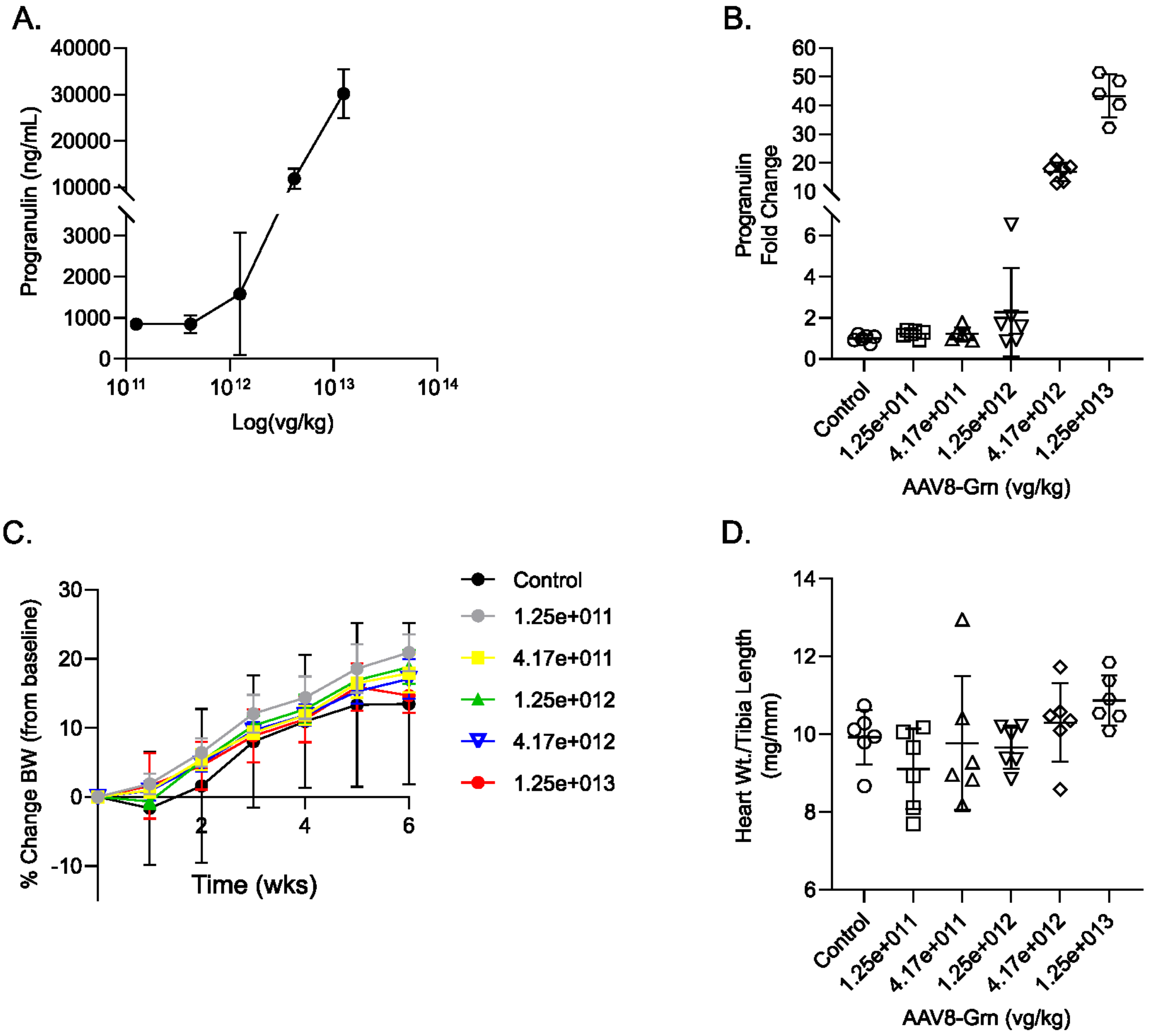

Supplemental Figure S1: AAV8-Grn bGH dose optimization. C57Bl/6N received vector at the doses indicated or equal volumes (100μL) of sterile saline via retro-orbital injections. 6-weeks post-injection mice were euthanized, and blood was collected by cardiac puncture. Plasma levels of progranulin were determined by ELISA as described in the methods. Plasma levels of progranulin increased in a dose-dependent manner **A**. Vector doses of $1.25x10^{12}$ and $4.17x10^{12}$ resulting in a 2.3-fold increase and 17-fold increase **B**. was chosen for subsequent studies. **C.** % change in body weight. **D.** Heart weight tibia length ratio.

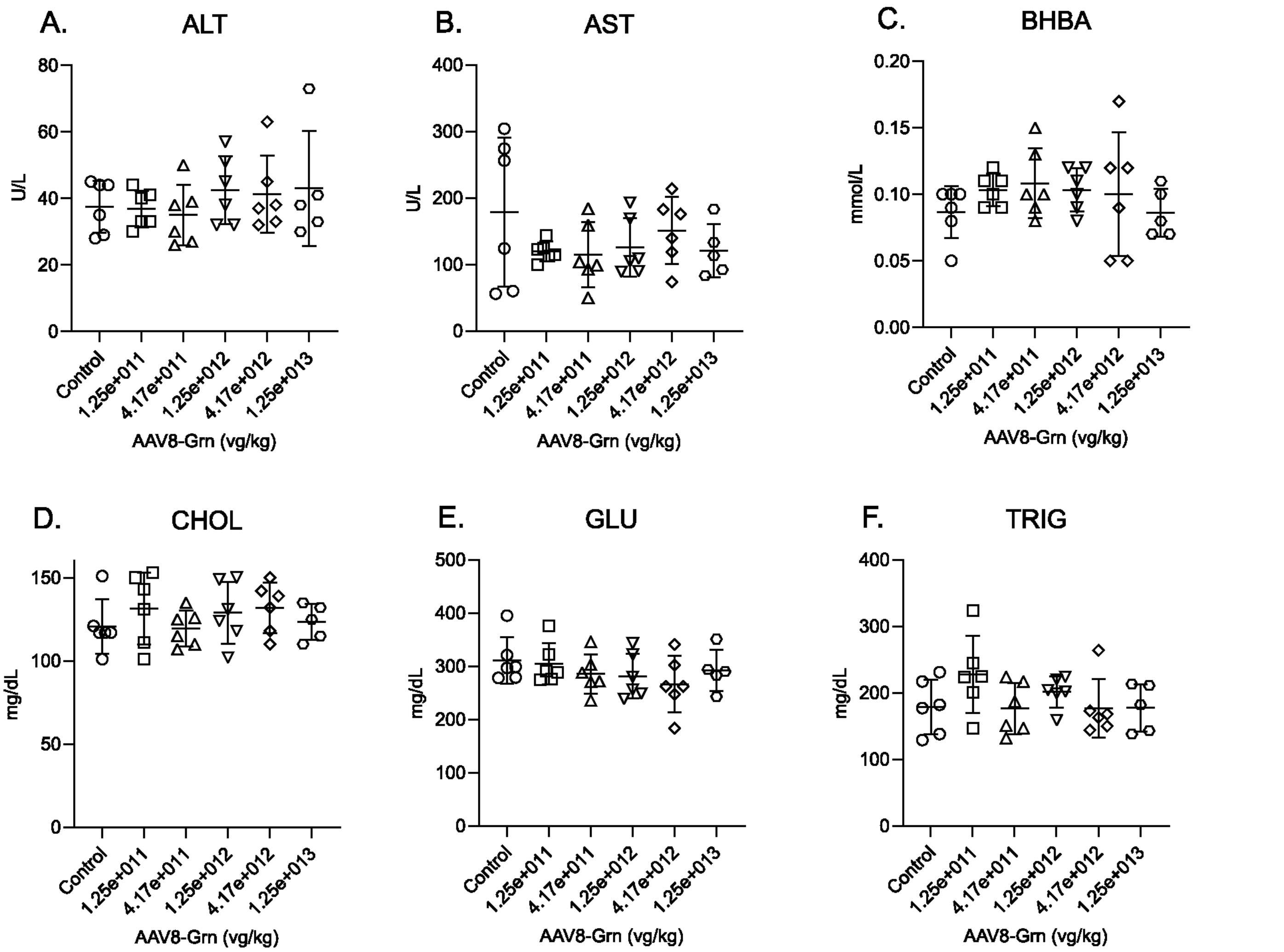

Supplemental Figure S2: Liver-directed overexpression of circulating progranulin with AAV8-Grn bGH does not cause liver toxicity or changes in plasma metabolic analytes. Plasma analytes were measured on the Siemens Advia clinical analyzer. **A.** ALT: alanine transaminase; **B.** AST: aspartate aminotransferase; **C.** BHBA: beta-hydroxybutyric acid; **D.** CHOL: cholesterol; **E.** GLU: glucose; **F.** TRIG: triglycerides.

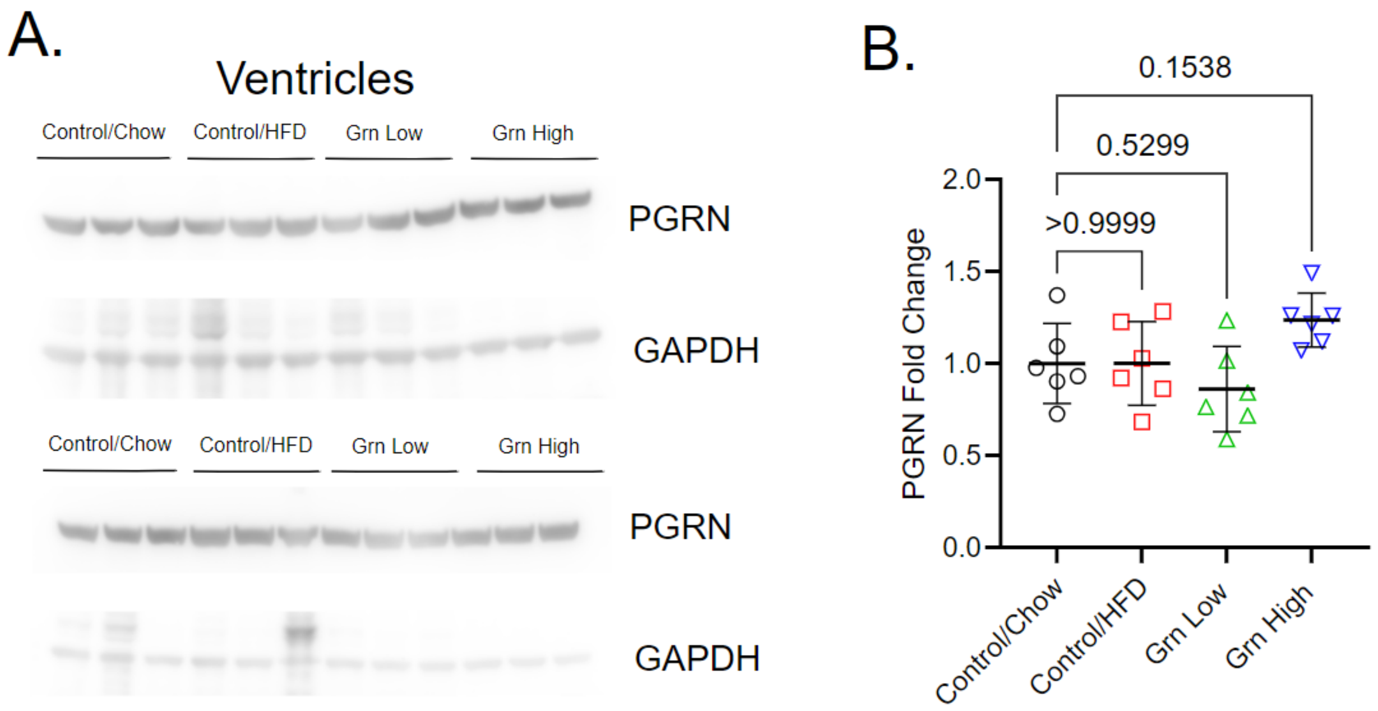

Supplemental Figure S3: Ventricle progranulin levels in mice overexpressing circulating progranulin. **A.** Western blots of progranulin protein in ventricular homogenates. **B.** Fold change of ventricular progranulin levels, normalized to GAPDH. Membranes were cut and incubated separately with PGRN and GAPDH primary antibodies. The original images and overlays displaying molecular weight markers are below.

Supplemental Fig. S3A (top, PGRN)

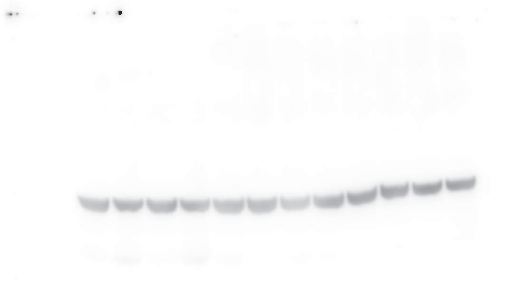

Supplemental Fig. S3A (top, PGRN)
Overlay with MWM.

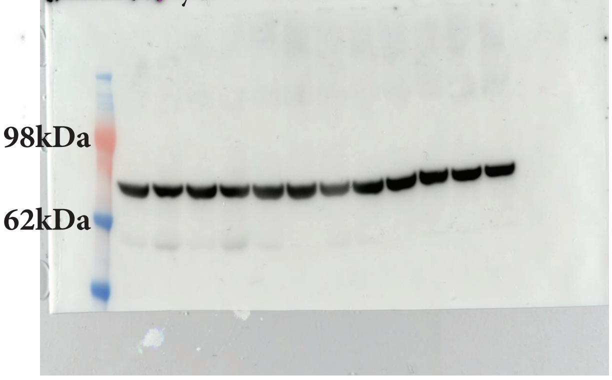

Supplemental Fig. S3A (top, GAPDH)

GAPDH-->

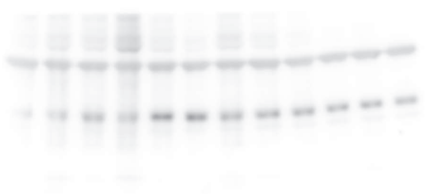

Supplemental Fig. S3A (top, GAPDH)
Overlay with MWM.

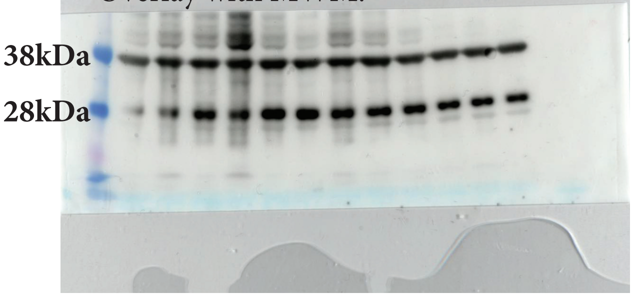

Supplemental Fig. S3A (Bottom, PGRN)

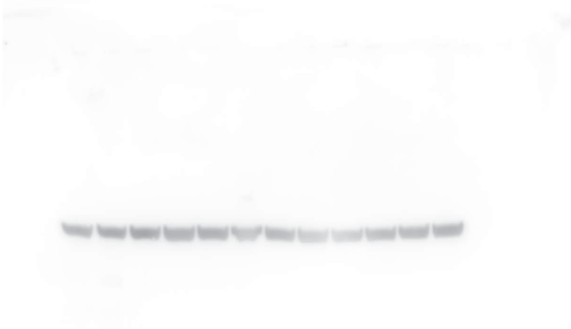

Supplemental Fig. S3A (Bottom, PGRN
Overlay with MWM.

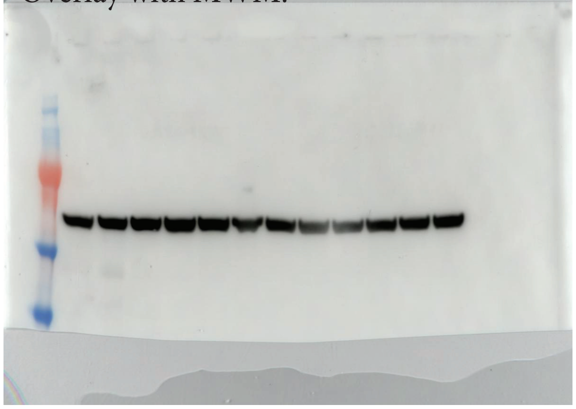

Supplemental Fig. S3A (Bottom, GAPDH)

GAPDH-->

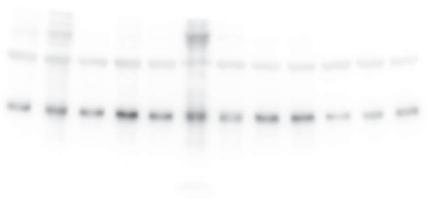

Supplemental Fig. S3A (Bottom, GAPDH)
Overlay with MWM.

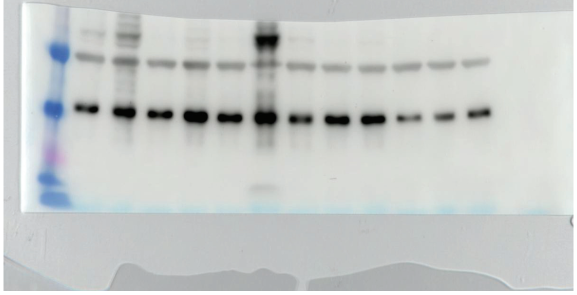

Supplement: Supplementary file 1 — Supplementary Information. [file 41598_2023_47311_MOESM1_ESM.pdf]
